# Concept and application of circulating proteasomes

**DOI:** 10.1038/s12276-021-00692-x

**Published:** 2021-10-27

**Authors:** Won Hoon Choi, Sumin Kim, Seoyoung Park, Min Jae Lee

**Affiliations:** 1 BK21 FOUR Biomedical Science Program, Seoul National University College of Medicine, Seoul, 03080 Korea; 2 Department of Biochemistry and Molecular Biology, Seoul National University College of Medicine, Seoul, 03080 Korea; 3 Department of Biomedical Sciences, Seoul National University Graduate School, Seoul, 03080 Korea

**Keywords:** Diagnostic markers, Proteasome

## Abstract

Proteostasis is primarily a function of protein synthesis and degradation. Although the components and processes involved in intracellular proteostasis have been studied extensively, it is apparent that extracellular proteostasis is equitably crucial for the viability of organisms. The 26S proteasome, a unique ATP-dependent proteolytic complex in eukaryotic cells, contributes to the majority of intracellular proteolysis. Accumulating evidence suggests the presence of intact 20S proteasomes in the circulatory system (c-proteasomes), and similar to other plasma proteins, the levels of these c-proteasomes may vary, potentially reflecting specific pathophysiological conditions. Under normal conditions, the concentration of c-proteasomes has been reported to be in the range of ~0.2–2 μg/mL, which is ~2–4-fold lower than that of functional plasma proteins but markedly higher than that of signaling proteins. The characterization of c-proteasomes, such as their origin, structure, role, and clearance, has been delayed mainly due to technical limitations. In this review, we summarize the current perspectives pertaining to c-proteasomes, focusing on the methodology, including our experimental understanding. We believe that once the pathological relevance of c-proteasomes is revealed, these unique components may be utilized in the diagnosis and prognosis of diverse human diseases.

## Disease biomarkers: Circulating protein complexes

Protein complexes called proteasomes degrade other proteins inside cells as part of cellular maintenance, but accumulating evidence suggests that proteasomes also circulate in the blood at levels that may vary in association with several diseases and also with normal aging. Researchers do not fully understand the cellular origins, biological functions and control of these circulating ‘c-proteasomes’, partly due to technical difficulties in analyzing their presence and their activity in blood samples. Min Jae Lee and colleagues at Seoul National University in South Korea review current understanding of c-proteasomes and their relevance to diseases including various cancers of blood cells and solid tissues, autoimmune disorders, sepsis and mild cognitive impairment. Emerging evidence suggests that c-proteasome levels and activity could serve to diagnose diseases and perhaps also to monitor and predict disease progression.

## Introduction: Proteasomes

Protein homeostasis, or proteostasis, is achieved both quantitatively (via translation and proteolysis) and qualitatively (by folding, cleavage, and posttranslational modification). In eukaryotic cells, the ubiquitin–proteasome system (UPS) is known to be involved in the degradation of the majority of regulatory proteins and the elimination of aberrant protein products, thereby critically contributing to the maintenance of the functional protein pool^1^. Polyubiquitylated UPS substrates are hydrolyzed by proteasomes in both the cytoplasm and nucleus into small peptide fragments. The 26S proteasome is an ~2.5 MDa holoenzyme comprising two distinct and dissociable components: the 20S catalytic particle and the 19S regulatory particle^2^. The cylindrical 20S proteasome is composed of four heteroheptameric rings (α_7_β_7_β_7_α_7_): the α_1−7_ outer ring forms a part of the substrate translocation channel, while the β_1−7_ inner ring retains the proteolytic active sites (caspase-like, trypsin-like, and chymotrypsin-like activities in the β1, β2, and β5 subunits, respectively). The 19S complex (with a molecular weight of ~930 kDa) recognizes, unfolds, and translocates the target substrates into the interior of the 20S proteasome (~730 kDa) and simultaneously deubiquitylates these substrates, thereby allowing ubiquitin recycling^3^. Marked advances in the structural and mechanical details of the 26S proteasome were recently made through cryo-electron microscopy^4–6^.

The archetype 20S proteasome was once considered a latent enzyme, partly because the gate in the substrate translocation channel was identified to be closed^7,8^. However, a growing number of endogenous substrates of the 20S proteasome with diverse physiological functions have been identified over the last decade, and now, this ubiquitin- and ATP-independent mode of proteasomal degradation is generally perceived as an alternative key regulator of the cellular proteome. Notably, the 20S proteasome degrades either fully or partially disordered proteins, including amyloid-β peptides^9^, tau^10,11^, α-synuclein^12,13^, and the cell-cycle-regulating proteins p21, p53, and p73^14–16^. In addition, oxidatively modified proteins, which largely affect the intracellular signaling pathway and cell viability when they accumulate, also form a class of 20S substrates^17,18^. This larger than anticipated physiological and pathological role of the 20S proteasome appears to be mediated by its conformational change upon engagement with substrates (from a resting form to a processing form)^19^. The Glickman group recently demonstrated that the binding of an unstructured model substrate was sufficient to induce conformational changes in α subunits and subsequently 20S gate opening, facilitating substrate hydrolysis without the involvement of the 19S proteasome or other activators^20^.

Several other chambered proteases with structural and mechanical similarities to the eukaryotic 20S proteasome have been identified in prokaryotes, which lack ubiquitin. For example, bacterial ClpP and HslV comprise 14 and 12 identical subunits, respectively, and sequester their active sites inside their gated chambers^21^. Direct binding of specific sequence motifs (in bacteria) or disordered regions (in eukaryotes) to chambered protease complexes may be evolutionarily analogous biochemical features in controlled proteolysis. Considering that more than 30% of the total proteins identified in eukaryotes are disordered-sregion-containing proteins^22^, the contribution of ubiquitin-independent 20S proteasome-mediated degradation in global proteostasis must be more prevalent than what is currently characterized. Fabre et al. used in vivo cross-linking prior to quantitative mass spectrometry to evaluate the steady-state ratio between the 20S and 26S proteasomes and found that 47–74% of these proteasomes exist in their free 20S form across a wide range of mammalian cell lines^23,24^. The ratio between the proteasome subtypes is expected to be dynamically switched due to changes in the cellular environment, allowing these proteasome subtypes to actively participate in cellular surveillance against pathological stress, such as reactive oxygen species^25–27^.

## Circulating proteasomes

For decades, proteostasis has commonly been referred to as an intracellular protein quality control mechanism. Our insight into how extracellular proteostasis operates and functions remains unclear, although more than 10% of the protein-encoding gene products are secreted^28^ and the majority of proteopathies, a group of human diseases associated with toxic protein accumulation and aggregation, occur in extracellular fluids^29^. Since the discovery of circulating proteasomes (hereafter referred to as c-proteasomes) in human blood ~30 years ago^30^, the clinical relevance of these components has been examined in various diseases, including myeloid and lymphoid malignancies, solid tumors, autoimmune disorders, sepsis, mild cognitive impairment, and other clinical conditions^31,32^. However, many of these studies used limited cohort sizes without further validation with independent reproduction. Here, we summarize the findings of previous studies on c-proteasomes (principally in chronological order) and discuss some of the key technical aspects of c-proteasome research. The availability of related reviews precludes the discussion on the extracellular proteasomes found in alveolar, cerebrospinal, and epididymal fluids, as well as other extracellular spaces^31,33^.

### c-Proteasomes in serum

The c-proteasome was first identified in the serum in 1993, not long after proteasomes were first purified from rabbit reticulocyte lysates^30,34^ (Table 1). Wada et al. used a customized enzyme-linked immunosorbent assay (ELISA) and self-raised monoclonal anti-α6 (then described as C2, the first proteasome subunit cloned in 1989^35^) antibody to evaluate the concentration of c-proteasomes in the serum of healthy individuals (*N* = 20) and patients. They determined that the average concentration of these proteins in the serum was 359.6 ng/mL and that these values were significantly elevated in patients with various hematological malignancies and in patients with liver disease (*N* = 175). They observed the highest c-proteasome level increase in patients with adult T cell leukemia (13.0 μg/mL, *N* = 6). They separated the serum proteins using gel filtration and enriched an ~650 kDa multimeric protein complex^30^, which was later characterized as the potential 20S proteasome. The levels of c-proteasomes in patients with various liver diseases were also elevated (1.34 μg/mL in patients with hepatocellular carcinoma; *N* = 16), and these values were positively correlated with those of serum alanine aminotransferase^30^.

**Table 1:** Level and activity of c-proteasomes in human serum.

First author	Year	Disease	Disease in detail	*N*	Serum c-proteasome concentration (ng/mL)	Chymotrypsin-like activity	Reference
Wada^a^	1993	Healthy		20	359.6	N/A	^30^
		Hematological malignancies	Acute leukemia	12	2900.4		
			Chronic myelogenous leukemia	7	1964.6		
			Myelodysplastic syndrome	3	1366.7		
			Non-Hodgkin’s lymphoma	16	866.3		
			Adult T cell leukemia	6	12,955.0		
			Multiple myeloma	12	577.8		
			Chronic lymphocytic leukemia	2	546.5		
			Waldenstrom’s macroglobulinemia	3	373.3		
		Liver diseases	Acute hepatitis	4	20,589.0		
			Chronic hepatitis	55	735,9		
			Liver cirrhosis	23	608.0		
			Hepatocellular carcinoma	16	1340		
			Fatty liver	16	577.9		
Egerer^a^	2002	Healthy		85	221.4	N/A	^36^
		Systemic autoimmune diseases	Autoimmune myositis	10	1598.4		
			Jo-1 syndrome	6	693.0		
			Systemic lupus erythematosus	76	681.3		
			Autoimmune hepatitis	37	669.8		
			Primary Sjögren syndrome	56	598.6		
			Antiphospholipid syndrome	11	565.9		
			Rheumatoid arthritis	66	531.6		
			Vasculitis	21	522.2		
			Systemic scleroderma	14	499.4		
			CREST syndrome	7	353.8		
			Myasthenia gravis	10	293.0		
Jakob^b^	2007	Healthy		50	224.1	N/A	^37^
		Multiple myeloma (MM)	Monoclonal gammopathy of undetermined significance	20	378.1		
			Total MM	141	599.6		
			Smoldering MM	40	314.7		
			Active MM	101	744.3		
Majetschak^b^	2008	Healthy		22	445.5	N/A	^38^
		Systemic autoimmune diseases	Connective tissue disease	35	831		
			Systemic lupus erythematous	56	889		
de Martino^b^	2012	Healthy		15	1520	N/A	^39^
		CRCC	Clear cell renal cell carcinoma (CRCC)	113	4660		
Roth^a^	2004	Healthy		15	2157	N/A	^40^
			Sepsis	15	33,551		
			Abdominal surgery	15	4661		
			Trauma in the intensive care unit	13	29,669		
Kakurina^b^	2017	Healthy		15	N/A	1150 U/ml	^41^
		HNSCC	Head and neck squamous cell carcinoma (HNSCC)	48	N/A	1166–1500 U/ml	

^a^The values are presented as the mean.

^**b**^The values are presented as the median.

It was not until the early 2000s that studies on c-proteasomes were reported again. Thereafter, the Feist group determined that the average concentration of c-proteasomes in the serum was 221.4 ng/mL in healthy individuals (*N* = 85), and they found that c-proteasome concentrations were significantly elevated, in a range of 300–700 ng/mL, in patients with a variety of systemic autoimmune diseases (total *N* = 314)^36^. Notably, they also detected 20S proteasome subunits in serum samples using ion-exchange chromatography, ammonium sulfate precipitation, and subsequent sucrose-gradient ultracentrifugation^36^. The serum c-proteasome levels in patients with multiple myeloma were found to be significantly elevated (median 744.3 ng/mL, *N* = 101) compared with those in healthy controls (median 224.1 ng/mL, *N* = 50)^37^. After chemotherapy, a significant decrease in the levels of c-proteasomes was observed in patients with a positive response to chemotherapy but not in nonresponders^37^.

Only a few other studies reported serum levels of c-proteasomes (Table 1). Several studies used an in-house ELISA and commercial anti-α6 antibodies to demonstrate that c-proteasome levels were significantly higher in patients with autoimmune diseases, such as systemic lupus erythematous (median 889 ng/mL [*N* = 56] vs. 446 ng/mL in controls [*N* = 22])^38^ and clear cell renal cell carcinoma (median 4.66 μg/mL [*N* = 113] vs. 1.52 μg/mL [*N* = 15] in controls)^39^. A dramatic increase in serum c-proteasome levels was observed in patients with sepsis resulting from peritonitis and pneumonia (33.6 μg/mL [*N* = 15] vs. 2.16 μg/mL [*N* = 15] in healthy controls)^40^. In addition to the c-proteasome concentration, the activity of the c-proteasomes in the sera was also evaluated and reported. These evaluations revealed that the activity of c-proteasomes was largely correlated with the size and degree of the differentiation of tumors in patients with head and neck squamous cell carcinoma (*N* = 48)^41^.

### c-Proteasomes in plasma

The majority of extracellular proteasome studies were performed using human plasma, usually using a small cohort (*N* ≤ 100). One of the earliest plasma c-proteasome studies included a total of 317 patients, with only 14–44 patients in each neoplastic disease group. The Bureau group performed an α6-targeting ELISA to evaluate the concentrations of c-proteasomes in the plasma of healthy individuals (2.36 μg/mL, *N* = 73) and demonstrated that these values were increased in patients with hemopoietic malignancies, including myeloproliferative disorder (4.10 μg/mL, *N* = 37) and myelodysplastic syndromes (2.92 μg/mL, *N* = 19). The c-proteasome levels were generally higher in plasma samples than in serum samples (Table 2). The changes in the plasma c-proteasome profile were correlated with the prognoses of some patients^42,43^. In a similar manner, the c-proteasome levels were measured in patients with metastatic malignant melanoma, and the highest c-proteasome levels were found to be correlated with the most advanced stages of melanoma (median 8.55 μg/mL in stage IV patients [*N* = 10] vs. 1.96 μg/mL in healthy individuals [*N* = 14])^44^. They also found that plasma c-proteasome levels were significantly higher in patients with hepatocellular carcinoma (3.74 μg/mL, *N* = 50) than in patients with cirrhosis without malignant transformation (1.81 μg/mL, *N* = 33) and controls (2.3 μg/mL, *N* = 40)^45^. In a follow-up study, the levels of c-proteasomes were significantly and positively correlated with the progression of melanoma, suggesting that these enzyme complexes could be used in the diagnosis of metastatic melanoma^46^.

**Table 2:** Level and activity of c-proteasomes in human plasma.

First author	Year	Disease	Disease in detail	*N*	Plasma proteasome concentration (ng/mL)	Chymotrypsin-like activity	Ref
Lavabre-Bertrand^a^	2001	Healthy		73	2356	N/A	^42^
		Hemopoietic malignancies	Solid tumor	20	7589		
			Myeloproliferative disorder	37	4099		
			Myelodysplastic syndromes	19	2922		
Stoebner^b^	2005	Healthy		14	1957	N/A	^44^
		Metastatic malignant melanoma	Stage I/II	13	2515		
			Stage III	6	3725		
			Stage IV	10	8554		
			Severe psoriasis	13	2981		
			Chronic idiopathic urticaria	6	3190		
Henry^b^	2009	Healthy		40	2302	N/A	^45^
		Liver cirrhosis	with hepatocellular carcinoma (HCC)	50	3737		
			without HCC	33	1808		
Henry^b^	2013	Metastatic melanoma	Stage I/II	53	184	N/A	^46^
			Stage III	41	228		
			Stage IV	27	499		
Ma^b^	2008	Healthy		40	N/A	0.80 pmol/s/mL	^47^
		Chronic lymphocytic leukemia		225	N/A	1.84 pmol/s/mL	
Ma^b^	2009	Healthy		97	N/A	0.8 pmol/s/mL	^48^
		Acute myeloid leukemia		174	N/A	2.0 pmol/s/mL	
		Myelodysplastic syndrome		52	N/A	1.4 pmol/s/mL	
Majetschak^b^	2010	Healthy		40	195	N/A	^54^
		Burn	Day 0	50	673		
			Day 30	40	116.5		
Heubner^b^	2011	Healthy		55	290	N/A	^55^
		Epithelial ovarian cancer	Patient	120	595		
			Patient, after therapy	68	457.5		
Hoffmann^b^	2011	Healthy		50	305	N/A	^56^
		Nonmetastasized breast cancer		224	397.5		
Fukasawa^b^	2015	Healthy		76	1,340	N/A	^57^
		Hemodialysis patient		76	1.381		
Manasanch^b^	2017	Multiple myeloma	Patient	45	N/A	0.83 pmol/s/ml	^58^
			After carfilzomib treatment		N/A	0.23 pmol/s/ml	
Oldziej^b^	2014	Healthy		30	2010	1.02 U/mg	^59^
		Multiple myeloma		64	4380	1.32 U/mg	

^a^The values are presented as the mean.

^b^The values are presented as the median.

The Albitar group demonstrated an increase in plasma c-proteasome activity in patients with chronic lymphocytic leukemia (*N* = 225)^47^, acute myeloid leukemia (*N* = 174), and advanced-stage myelodysplastic syndrome (*N* = 52)^48^. All three proteolytic activities in patients were found to be elevated compared with those in healthy controls, and they showed significant correlations with prognosis, therapeutic response, and survival prediction. In both cases, the plasma samples (collected in EDTA tubes) were incubated with a 1% final concentration of sodium dodecyl sulfate (SDS) for 15 min to “activate” the plasma^47,48^. To clinically employ c-proteasome activity as a reliable biomarker, it seems to be important to simultaneously quantify the c-proteasome absolute (rather than relative) activity and concentration.

**Fig. 1:**
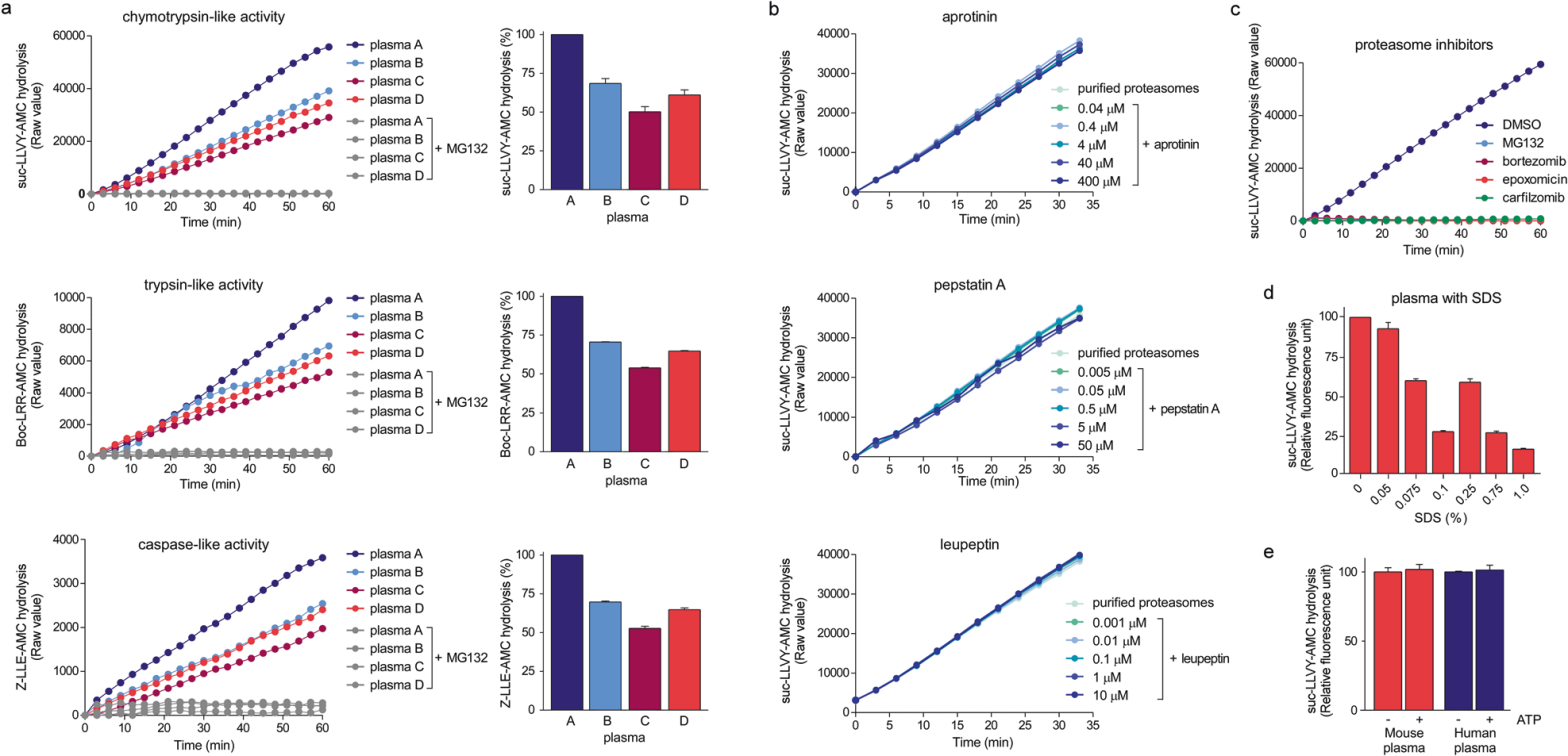
Activity of circulating proteasomes (c-proteasomes) in human plasma. **a** Plasma samples were collected from four individuals (plasma A–D) in EDTA tubes, and their c-proteasome activity (in 20 μL of plasma) was evaluated by monitoring the hydrolysis of the fluorogenic reporter substrates (final concentration of 250 μM in a total of 100 μL reaction), such as suc-LLVY-AMC (for chymotrypsin-like activity), Boc-LRR-AMC (for trypsin-like activity), and Z-LLE-AMC (for caspase-like activity) in the presence or absence of the proteasome inhibitor MG132 (10 μM). These reactions were performed using assay buffer (50 mM Tris-HCl [pH 7.5], 1 mM EDTA, 1 mg/mL BSA, 1 mM ATP, and 1 mM DTT). Sodium dodecyl sulfate (SDS) was not added to the reaction unless otherwise described. The graphs (left) represent the results obtained in three independent experiments, and the mean of the raw fluorescence values (right) at 60 min are plotted with their standard deviations (*N* = 3). **b** Human c-proteasome activity was analyzed using suc-LLVY-AMC as the substrate, along with a wide range of protease inhibitors, including aprotinin (trypsin inhibitor), pepstatin A (aspartyl protease inhibitor), and leupeptin (serine/cysteine protease inhibitor). **c** As in (**b**), but using different proteasome inhibitors (10 μM MG132, 2 μM bortezomib, 2 μM epoxomicin, or 100 nM carfilzomib). **d** The plasma samples were preincubated with SDS at the indicated final concentrations for 10 min before initiating the suc-LLVY-AMC hydrolysis reactions. Relative fluorescence values after 30-min reactions were normalized to those obtained in the presence of 10 μM MG132. The values represent the mean ± standard deviation (*N* = 3). **e** As in (**a**), except that assay buffer without ATP was used. No significant changes were observed.

The hydrolysis of peptidyl fluorogenic substrates has been widely used to determine c-proteasome activity in human blood. These substrates include succinyl-Leu-Leu-Leu-Val-Tyr-7-amino-4-methylcoumarin (suc-LLVY-AMC), ter-butyloxycarbonyl-Leu-Arg-Arg-AMC (Boc-LRR-AMC), and carbonylbenzyl-Leu-Leu-Glu-AMC (Z-LLE-AMC), which are specific for chymotrypsin-like β5, trypsin-like β2, and caspase-like β1 activities, respectively, in the 20S catalytic chamber^49^. The fluorescence intensities must be normalized to the values obtained in the presence of proteasome inhibitors as the identical principle applies to assess the purified proteasomes and whole-cell/tissue lysates. The hydrolysis of suc-LLVY-AMC, which was used as a primary substrate during the first proteasome assays in the late 1980s^34^, is largely regarded to represent the overall proteasome activity^50^. Using human plasma, we also observed that the changes in the activity of the three catalytic sites in c-proteasomes were highly correlated (Fig. 1a). In addition, suc-LLVY-AMC hydrolysis was not affected by a number of protease inhibitors; however, it was effectively abolished by reversible or irreversible proteasome inhibitors (Fig. 1b, c). In vitro reconstituted polyubiquitylated proteins, such as polyubiquitylated sic1 or DHFR^51,52^, which act as more physiologically relevant substrates for proteasomal degradation, have rarely been evaluated in studies related to c-proteasome activity.

A critical concern for the assessment of c-proteasome activity using human blood and fluorogenic substrates is the addition of varying concentrations of SDS (0.1–1.0% final concentration), which is intended for the “preactivation” of c-proteasomes. Although mildly elevated proteasome activity after SDS incubation was reported^34,53^, it has also been shown that the 20S component exhibits strong suc-LLVY-AMC hydrolysis activity even in the absence of SDS and that a high concentration of SDS may inadvertently inhibit c-proteasome activity (Fig. 1d). It is possible that certain concentrations of SDS may result in the dissolution of phospholipid membrane-like extracellular vesicles encapsulating c-proteasomes, leading to an increase in c-proteasome activity in these assays. Therefore, the application of SDS to mediate c-proteasome activation requires further validation. In addition, we found that the plasma samples prepared in EDTA tubes showed much higher c-proteasome activity than those prepared in heparin tubes or the serum samples that underwent a clotting process (data not shown). The exact mechanism underlying these differences remains unclear, but it seems to be critical to resolve this issue to establish a standardized protocol and improve experimental reproducibility.

c-Proteasome levels and activities were both found to be elevated in burn patients on the day of admission compared to those in healthy volunteers^54^. The highest median 20S concentration (673 ng/mL, *N* = 50) was reported on Day 0, but the levels gradually decreased within the first week following burn injury, eventually returning to baseline levels (195 ng/mL, *N* = 40) after 30 days. The 26S form of c-proteasomes was virtually undetectable in the ELISA-based assay^54^. A standard proteasome peptidase assay was performed using the plasma samples collected in EDTA or sodium citrate tubes: 100 μM suc-LLVY-AMC and 35 μL of plasma in 10 mM Tris-HCl (pH 7.5) buffer at 37 °C for 60 min, without SDS activation. Basal proteasome activity was determined upon the addition of an irreversible proteasome inhibitor, epoxomicin (7 μM), to the mixture. The relatively weak c-proteasome activity was detected in their samples: among the nine randomly selected samples, only three specimens exhibited detectable fluorescence signals corresponding to the reporter peptides^54^. Nevertheless, the findings of this study strongly supported the notion that c-proteasomes are primarily expressed in the 20S form and are enzymatically active even without further activation.

In a large-scale study, the Heubner group observed elevated plasma levels of c-proteasomes in patients with epithelial ovarian cancer (595 ng/mL, *N* = 120) and nonmetastatic breast cancer (397.5 ng/mL, *N* = 224) compared to the levels in healthy controls (290 ng/mL, *N* = 55)^55,56^. Fukasawa et al. thoroughly assessed plasma c-proteasome levels and various other clinical parameters in 76 patients undergoing hemodialysis and found a significant negative correlation between c-proteasome levels and abdominal muscle area^57^. In patients with multiple myeloma, treatment with a single dose of the proteasome inhibitor carfilzomib (20 mg/m^2^), which irreversibly targets the β5 (chymotrypsin-like) proteolytic site, led to drastically reduced chymotrypsin-like activity, but it did not affect caspase- or trypsin-like activities^58^. In a similar study conducted by Oldziej et al.^59^, both the concentration and the activity of c-proteasomes in plasma samples were shown to be significantly higher in patients with multiple myeloma (4.38 μg/mL and 1.32 U/mg [*N* = 64]) than in healthy controls (2.01 μg/mL and 1.02 U/mg [*N* = 30]). The Matuszczak group analyzed the plasma levels of c-proteasomes in pediatric patients with mild head injury^60^, acute appendectomy^61^, and moderate to major burns^62^. In general, the group found that c-proteasome activity and concentration increased initially following an acute onset and reduced gradually after treatment. Most recently, a significant correlation was reported between the c-proteasome levels and the decline in the lean tissue indices in hemodialysis patients over two years, although c-proteasome levels could not be used to predict patient survival in that particular time period^63^.

A series of biochemical purifications, including albumin removal, ammonium sulfate precipitation, anion exchange column fractionation, and affinity purification, followed by negative-staining electron microscopy, revealed that most c-proteasomes in the plasma exist in the 20S form^64^. ATP-independent proteolytic activity of c-proteasomes was observed in both mouse and human plasma, consistent with the general principle of the 20 S c-proteasomes established in previous studies (Fig. 1e). Considering the ATP-depleted environment of the blood and the role of ATP in the association between the 20S and 19S subunits, it is conceivable that c-proteasomes circulate in the free 20S form in human plasma. This possibility would suggest a protective function of 20S c-proteasomes in the clearance of potentially harmful misfolded proteins in the extracellular space, which is highly oxidizing (in comparison with the reducing nature of the intracellular compartment). We postulate that the c-proteasomes in the plasma may exhibit different enzymatic activities than those exhibited by intracellular proteasomes. For example, 20S c-proteasomes with limited proteolytic activity may catalyze the partial cleavage of disordered (or oxidized) plasma proteins, similar to the function of the serine proteases involved in hemostasis. Certain translation initiation factors, transcription factors, and heat shock proteins have already been described to be endoproteolytically processed by intracellular proteasomes^65^. Therefore, the physiological role of the c-proteasomes in extracellular proteostasis may be to assist in untangling the aggregation-prone proteins (rather than complete protein degradation) and in facilitating their cell-surface receptor-mediated lysosomal degradation.

## Conclusions and clinical perspectives

In this review, we have summarized the current knowledge of c-proteasomes in the serum and plasma, focusing on methodological aspects. Many studies discussed herein reported a strong correlation between disease status and the level (or activity) of 20S c-proteasomes. However, c-proteasomes are still an orphan biological entity for which the origin, function, substrate, and regulatory mechanism have yet to be elucidated. Our understanding of the potential of c-proteasomes as diagnostic and prognostic factors at the cellular level is far from comprehensive. The delays in this field are primarily attributed to technical issues associated with blood chemistry and the lack of a standardized protocol. The implementation of a wide variety of laboratory procedures, from sample acquisition procedures to assay protocols, also makes it difficult to obtain consistency and concordance between the results of independent c-proteasome studies. Since researchers are reluctant to publish negative or nonsignificant findings, a possible explanation for the very small number of total c-proteasome studies (approximately one paper per year) might be attributed to the negative outcomes in the unreported studies.

Despite many unresolved issues around c-proteasomes, we believe that it is still possible to establish a validated biomarker based on c-proteasomes by improving both research and clinical practices. Biomarkers of Alzheimer’s disease may serve as a good example: only a few years ago, the development of these markers was regarded practically unachievable, but they are now cross-validated and almost ready for clinical implementation due to biological and technical advances^66^. Considering that cellular proteasome activity has been found to decrease with age^67^, it seems interesting to examine whether the changes in c-proteasome activity are correlated with the aging process. It seems possible that insufficient renal function, such as low urinary filtration, could lead to elevated levels of c-proteasomes. Notably, the assessment of the enzymatic activity of c-proteasomes is much less expensive (in the cent range) and faster (<30 min per assay) than the performance of antibody-based methodologies. At present, the activity of c-proteasomes can be considered to be positively correlated with their plasma concentration in humans^32^, and most FDA-approved blood tests are decades old. Therefore, slight advancements in c-proteasome biology and the technology for monitoring c-proteasome activity may fundamentally transform the procedural approaches and allow broader applications of c-proteasomes in routine clinical evaluations in the near future.

**Fig. 2:**
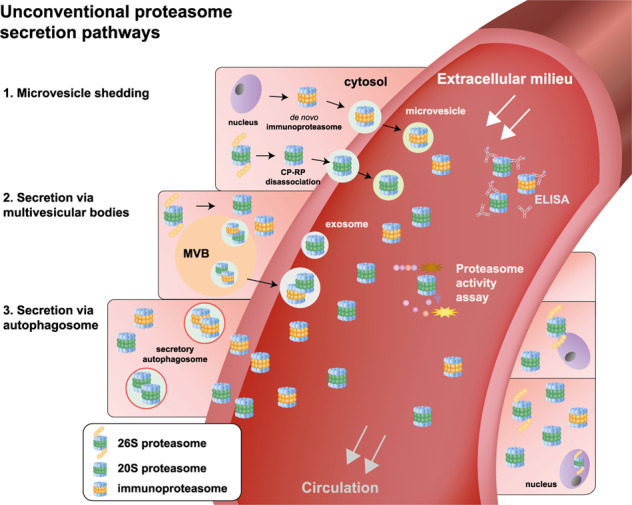
Schematic representation of proteasome secretory pathways. Unlike classic protein secretion involving ER-to-Golgi transport, cytosolic proteasomes are expected to be directed to the extracellular space through unconventional secretory pathways. Under stress or pathological conditions, the 26S proteasome dissociates into 20S and 19S particles, and simultaneously, de novo synthesis of the 20S immunoproteasome may be induced. The 20S forms of proteasomes can be released through microvesicle shedding (route 1). Alternatively, the 20S forms of proteasomes are first packaged into endocytic compartments, such as multivesicular bodies and autophagosomes (routes 2 and 3, respectively), which later fuse with the plasma membrane and are then released as exosomes. The level and activity of 20S c-proteasomes can be readily detected using ELISA and fluorogenic reporter peptides, respectively. The physiological and pathological significance of these pathways remains to be investigated.

The origin of c-proteasomes is one of the major unresolved questions in c-proteasome research. While some studies support the notion that c-proteasomes are passively released from ruptured cells, others suggest that c-proteasomes are transported into the blood via an active secretory mechanism^31,33^ (references therein). It seems plausible that the 20S proteasome, which is ~10 nm long, is packaged into a membrane-bound organelle and released in a 30–100 nm extracellular vesicle such as exosomes (Fig. 2; route 2). In cultured T lymphocytes, significant numbers of proteasomes are reported to be secreted into the media via an exosomal pathway^68,69^. We hypothesize that the encapsulated 20S proteasomes originate from not only dissociation from the 19S complex but also de novo synthesis (and assembly) of 20S immunoproteasomes under stress conditions. Alternatively, 20S proteasomes may be shed into the extracellular spaces via microvesicles (Fig. 2; route 1), as the Vidal group recently identified^70^ or the autophagy–lysosome system may affect the quantity and quality of extracellular components through autophagosome-mediated unconventional secretion (Fig. 2; route 3) instead of the turnover of intracellular proteasomes^71,72^. Once this information is available, we may have a better biological framework to study the subunit composition, subtypes, and, most of all, the functional relevance of c-proteasomes. Because the concentration of proteasomes is >100-fold higher in the cytosol than in the extracellular milieu^73^, pharmacological modulations of their secretion system may result in dynamic alterations in the concentration of c-proteasomes in human blood, thereby affecting the pathophysiology of proteopathies characterized by the deposition of extracellular proteins.
